# Vaccinating for natural killer cell effector functions

**DOI:** 10.1002/cti2.1010

**Published:** 2018-01-31

**Authors:** Helen R Wagstaffe, Jason P Mooney, Eleanor M Riley, Martin R Goodier

**Affiliations:** ^1^ Department of Immunology and Infection London School of Hygiene and Tropical Medicine London UK; ^2^ The Roslin Institute and Royal (Dick) School of Veterinary Studies University of Edinburgh Midlothian UK

**Keywords:** accessory cell, cytokines, NK cell, vaccination

## Abstract

Vaccination has proved to be highly effective in reducing global mortality and eliminating infectious diseases. Building on this success will depend on the development of new and improved vaccines, new methods to determine efficacy and optimum dosing and new or refined adjuvant systems. NK cells are innate lymphoid cells that respond rapidly during primary infection but also have adaptive characteristics enabling them to integrate innate and acquired immune responses. NK cells are activated after vaccination against pathogens including influenza, yellow fever and tuberculosis, and their subsequent maturation, proliferation and effector function is dependent on myeloid accessory cell‐derived cytokines such as IL‐12, IL‐18 and type I interferons. Activation of antigen‐presenting cells by live attenuated or whole inactivated vaccines, or by the use of adjuvants, leads to enhanced and sustained NK cell activity, which in turn contributes to T cell recruitment and memory cell formation. This review explores the role of cytokine‐activated NK cells as vaccine‐induced effector cells and in recall responses and their potential contribution to vaccine and adjuvant development.

## Introduction

Vaccination is a cost‐effective way of reducing the burden of, eliminating and – in exceptional cases – eradicating infectious diseases. Whilst a number of effective vaccines are currently licenced, many highly prevalent and complex diseases remain without effective prophylactic vaccines. Protective titres of neutralising antibodies and expanded populations of effector and memory B and T lymphocytes are viewed as measures of protection for many vaccines. Currently, the generation of durable antigen‐specific memory forms the basis of vaccine development and evaluation of vaccine efficacy.^1^, ^2^ Developing vaccines to overcome pathogen polymorphism and complexity demands new approaches to vaccine design and evaluation; this in turn requires the identification of novel correlates of protection and determination of optimal dosing schedules. The activation of Natural Killer (NK) cells represents a potential route for further optimisation of vaccination strategies by harnessing their role as antipathogen effector cells which integrate innate and acquired immune responses.

NK cells are large, granular, type 1 lymphoid cells that express a wide variety of germline‐encoded receptors on their surface. Direct NK cell activation is controlled by the balance between signals transduced by inhibitory and activating receptors; NK cells are also activated indirectly by innate cytokines such as type I interferons (IFN), IL‐12, IL‐15 and IL‐18, released from accessory cells.^3^ Because NK cells do not rearrange receptors to permit antigen‐specific recognition, they are normally classified as cells of the innate immune system. NK cells are among the first cells to respond during primary infection and contribute to the early control of viral infections including herpesviruses and influenza infections.^4^, ^5^, ^6^, ^7^, ^8^ However, NK cells can also augment and shape the subsequent adaptive response by secretion of cytokines (including IFN‐γ) and chemokines (such as IP‐10, MIP‐1α and MIP‐1β), reducing viral loads by killing infected cells, inhibiting viral entry and replication by production of chemokines which compete for human immunodeficiency virus (HIV) coreceptor CCR5^9^ and by controlling expansion of the CD4^+^ T cell compartment.^10^, ^11^ In turn, the secondary (adaptive) immune response can activate NK cells via secretion of cytokines such as IL‐2 from CD4^+^ T cells and via FcγRIIIa (CD16)‐dependent recognition of antigen‐antibody complexes.^12^, ^13^, ^14^

Several studies have shown that NK cells can acquire some features of adaptive lymphocytes, raising the possibility that the memory‐like properties of these cells could be induced or enhanced by vaccination. Early examples of NK cell adaptive features arose from mouse studies of murine cytomegalovirus (MCMV) and hapten‐induced contact hypersensitivity. The MCMV viral protein m157 on the surface of infected cells was shown to recognise NK cell Ly49H receptor and leads to clonal expansion of effector NK cells and generation of a pool of self‐renewing m157‐responsive NK cells; these cells respond robustly to subsequent MCMV infection when transferred to naive mice.^15^ NK cells from Rag2^−/−^ mice were shown to transfer hapten‐specific memory‐like responses (lasting up to 4 weeks) to naive mice despite the absence of T and B cell immunity.^16^ More recently, virus antigen‐specific NK cell killing has been reported in rhesus macaques,^17^ and there are suggestions of pathogen‐specific responses among human NK cells. These include influenza hemagglutinin (HA) antigen recognition by NK cell NKp46 and HLA‐E stabilisation by HCMV peptides for recognition by NK cell CD94/NKG2C heterodimers.^18^, ^19^, ^20^

An increasing number of studies in humans demonstrate activation of NK cells during recall responses to pathogens in vaccinated subjects. *In vitro* NK cell responses to components of the DTP vaccine (diphtheria toxoid, tetanus toxoid and whole cell inactivated pertussis), Bacille Calmette–Guérin (BCG) and influenza vaccine are enhanced after vaccination^14^, ^21^, ^22^, ^23^ and heightened NK cell IFN‐γ and degranulation responses have been detected after vaccination against rabies.^24^ In contrast to the memory responses described above, these postvaccination responses are dependent on vaccine‐specific CD4^+^ memory T cells and, in particular, their rapid secretion of IL‐2.^23^, ^24^

Although the ‘antigen‐specificity’ of these postvaccination NK cell responses resides in the CD4^+^ T cell pool, the NK cells are also modified as a result of vaccination. Innate cytokines, which can be induced by live or killed whole pathogen vaccines or by adjuvants, are potent NK cell activators and can induce their differentiation into cytokine‐induced memory‐like (CIML) NK cells. First described as being generated by cytokine coculture *in vitro,* CIML NK cells have an enhanced ability to secrete IFN‐γ and become cytotoxic in response to cytokine and MHC‐devoid K562 cell restimulation for up to 21 days after the initial stimulation.^13^, ^25^, ^26^, ^27^ *In vitro* cytokine activation with IL‐18 and IL‐12 and/or IL‐15 induces expression of CD25, thereby generating CIML NK cells with enhanced responsiveness (demonstrated by IFN‐γ production and cytotoxicity) to picomolar concentrations of IL‐2.^28^ More importantly perhaps, CIML NK cells can be induced by vaccination in response to CD4^+^ T cell‐derived IL‐2 and myeloid cell‐derived IL‐12 and type I interferons, and have been implicated in the enhancement of NK cell function *ex vivo*.^13^, ^24^, ^29^, ^30^, ^31^, ^32^, ^33^ Vaccination‐induced CIML NK cells can be detected for up to 12 weeks postvaccination in European subjects^13^ and up to 6 months in west African vaccines.^33^ Table 1 summarises the evidence for vaccination‐induced CIML NK cells.

**Table 1:** Evidence of induction of human cytokine‐induced memory‐like (CIML) NK cells by vaccination

Pathogen	Host species	Vaccination	Increased responsiveness *in vitro*	Duration of response	References
Influenza	Human	TIV	IL‐12, IL‐15, IL‐18	3 months (UK) or 6 months (Gambia)	Goodier *et al*.^60^ and Darboe *et al*.^33^
YFV	Human	YF‐17D	IL‐12	15 days	Marquardt *et al*.^30^
SIV	Macaque	Ad26 HIV‐1, DNA‐Ad26	IL‐12, IL‐15	38 weeks	Vargas‐Inchaustegui *et al*.^77^
TB	Human	BCG	IL‐12, IL‐18	ND	Suliman *et al*.^29^

ND, not determined.

Immature CD56^bright^ and CD56^dim^CD57^−^ NK cells are more responsive to exogenous cytokines and produce more IFN‐γ than do the more mature, predominantly cytotoxic, CD56^dim^CD57^+^ NK cell subset.^34^ Accordingly, vaccination‐induced CIML NK cells are restricted to the less differentiated subsets of NK cells and their induction is accompanied by proliferative expansion of the least mature CD56^bright^ NK cells and CD56^dim^CD57^−^NKG2C^−/+^ subsets.^13^, ^33^ Enrichment of less differentiated NK cells in lymph nodes and effector tissues could influence the impact of CIML NK cells induced by vaccination. The highest proportion of human immature CD56^bright^ (CD16^−^) NK cells are found in the lymph nodes^12^, ^35^ and produce IFN‐γ in response to CD4^+^ T cell‐derived IL‐2, thereby potentially influencing subsequent adaptive responses.^12^ A higher percentage of adoptively transferred pre‐activated CIML NK cells were found in the lymph nodes of mice compared to control NK cells^25^ and localised in the bone marrow, spleen and blood of mice and in the bone marrow of patients with acute myeloid leukaemia.^27^ The tissue localisation of NK cells amenable to cytokine‐mediated pre‐activation may also be crucial to functional outcomes. Human liver, in contrast to secondary lymphoid tissues, is enriched for resident CD56^bright^ NK cells with high natural cytotoxicity receptor and NKG2D expression, strong target cell‐mediated degranulation but poor IFN‐γ production.^36^ Tissue‐resident innate lymphoid cells (ILC) which are phenotypically distinct from NK cells may, however, also be sensitive to pre‐activation by vaccine‐induced cytokines. Murine liver ILC‐1, for example, is highly sensitive to IL‐12 stimulation and produces more IFN‐γ at the sites of MCMV infection.^37^ When taken together with the emerging literature on the impact of persistent viral infections (such as human cytomegalovirus infection (HCMV); see below) on NK cell function,^14^ it is possible that differences between or within human populations in proportions of CIML NK cells (due to differences in recent infection and vaccination history) may contribute to differences in vaccine immunogenicity and effectiveness.^33^, ^38^, ^39^

Expanded populations of highly differentiated NK cells in individuals chronically infected with HCMV were first described more than a decade ago.^40^ Many of these highly differentiated cells were subsequently shown to have undergone key intrinsic changes such as the loss of the signalling molecules FcεRγ, SYK and EAT‐2, associated with stable epigenetic changes in the promotor regions of genes involved in NK cell function, including IFN‐γ.^41^, ^42^, ^43^ These ‘adaptive’ NK cells display enhanced antibody‐dependent cellular cytotoxicity (ADCC) activity towards HCMV‐infected target cells suggesting they are specialised for controlling virus reinfection or reactivation and antigen‐specific.^44^, ^45^ However, despite the likely dominance of such adaptive cells in populations with endemic HCMV infection, the generation of CIML from less differentiated NK cells persists after vaccination^33^ (reviewed in ref. 46). It appears, therefore, that there is a balance of CIML and highly differentiated NK cell effectors which may be altered by vaccination. Less differentiated NK cells are shorter‐lived, possess higher levels of cytokine receptors and higher intrinsic proliferative capacity; vaccination may simply contribute to the homoeostatic maintenance of these cells. The benefits of preferentially expanding and generating CIML NK cells from these subsets are unknown but could be more functionally significant in young infants where highly differentiated cytotoxic effectors are lacking.^47^ On the other hand, loss of IL‐12 responsiveness and independence of this cytokine for IFN‐γ production is a well‐known feature of more differentiated NK cell effectors; more focused antibody‐driven responses may be advantageous in restricting the potential for inflammation associated damage in older individuals.

In the remainder of this review, we explore in more detail the potential role of NK cells, activated by myeloid cell‐derived cytokines or by components of adaptive immunity (CD4^+^ T cell IL‐2 or pathogen‐specific antibodies), as effectors of vaccination against a number of globally important infectious diseases.

## Influenza

Seasonal influenza epidemics result in 3–5 million cases of influenza globally and up to half a million deaths every year as well as putting intolerable pressure on health systems and causing major economic losses.^48^ Annual variation in the predominant circulating strains of influenza viruses mitigates vaccine‐induced or naturally acquired cross‐protective immunity, necessitating annual revaccination of high‐risk groups (pregnant women, children of 6 months to 5 years and the elderly).^48^ A cross‐protective ‘universal’ influenza vaccine is a major priority for influenza vaccine development.

Influenza virus induces secretion of innate cytokines (including IFN‐α, IL‐12 and IL‐18) from accessory cells such as macrophages and dendritic cells (DCs); in turn, these cytokines support the very rapid activation of NK cells (within hours of infection).^49^, ^50^ These activated NK cells are cytotoxic, secrete IFN‐γ and upregulate cytokine receptors such as CD25 (IL‐2Rα)^51^ and can reciprocally activate DCs, thereby promoting T cell recruitment to sites of infection and to lymph nodes.^52^ *In vitro* restimulation of peripheral blood mononuclear cells (PBMC) from trivalent influenza vaccine (TIV)‐vaccinated volunteers with inactivated influenza virus induces much higher frequencies of IFN‐γ producing and degranulating NK cells compared to restimulation of prevaccination PBMC from the same people.^13^, ^18^, ^23^, ^53^ The heightened NK cell response becomes evident as early as 2 weeks postvaccination but is normally lost by 12 weeks. Postvaccination enhancement of NK cell IFN‐γ production was dependent on IL‐2 produced from CD4^+^ T cells, whilst degranulation responses were dependent on IL‐2 and on the presence of anti‐influenza antibody.^13^, ^23^ A costimulatory role for innate myeloid cell‐derived cytokines was also demonstrated by partial inhibition of TIV restimulation responses with IL‐12, IL‐18 and IFN‐αβR2 blockade.^13^

Indeed, consistent with the generation of CIML NK cells, antigen‐independent *in vitro* responses to exogenous IL‐12 and IL‐18 were also elevated for up to 3 months after influenza vaccination in a UK study,^13^ but this response was detected for up to 6 months in African subjects.^33^ Enhancement of NK cell responses after influenza vaccination is therefore mediated by indirect mechanisms involving antigen‐specific cellular CD4^+^ and humoral responses combined with a shorter‐lived CIML component. Such enhanced NK cell function after seasonal influenza vaccination may contribute to protective immunity to influenza, but, given the dependence on antigen‐specific T cells and antibodies, does not in itself overcome the need for regular revaccination. However, the search for a ‘universal influenza vaccine’ has identified the conserved ‘stalk’ of the polymorphic HA molecule^54^ and other nonvaccine antigens^55^ as possible targets of broadly neutralising antibodies which mediate ADCC.^56^, ^57^ Stalk‐specific antibodies that mediate NK cell ADCC are present after natural infection and after vaccination with TIV or monovalent adjuvanted H1N1^58^ and nucleoprotein (NP)‐specific ADCC‐mediating antibodies induced by seasonal influenza vaccination demonstrate cross‐reactivity with H7N9 avian influenza NP.^59^ As mature CD56^dim^CD57^+^ NK cells and HCMV‐induced ‘adaptive’ NK cells are both potent mediators of ADCC and preferentially respond to influenza antigens after vaccination,^60^ NK cells may be of particular importance as effectors of the next generation of universal influenza vaccines.

## Yellow fever

The live attenuated yellow fever virus (YFV) vaccine 17D is one of the most effective vaccines developed to date; 99% of recipients are protected for more than 10 years after a single vaccination.^61^ For this reason, YF‐17D has been used as a tool to identify highly effective early (innate) immune responses to acute viral infection in humans.^30^, ^62^ YFV infects and induces TLR‐mediated signalling in hepatocytes and cells of the innate immune system such as monocytes and DCs. In mouse models of YFV infection or YF‐17D vaccination, NK cells accumulate in the spleen and are major producers of IFN‐γ.^63^, ^64^ Induction of innate cytokines such as IL‐1α and chemokine IP‐10 (CXCL10), and upregulation of the early activation and proliferation markers CD69 and Ki‐67 on NK cells are detected as early as 3 days postvaccination in humans.^30^, ^62^, ^65^ NK cell activation peaks at the same time as viral load, 6 days postvaccination and correlates directly with a rise in plasma type I and type III interferons. Thereafter, viral load and NK cell responses decline rapidly returning to baseline by day 10 and 15 postvaccination, respectively.^30^, ^65^

In a study in Uganda, pre‐existing IFN‐γ producing NK cells in an activated immune microenvironment were associated with lower viral loads and subsequently reduced antibody titres after YF‐17D vaccination.^38^ NK cell IFN‐γ responses to YFV correlated with increased *in vitro* responsiveness of less differentiated NK cells to innate cytokines such as IL‐12 after vaccination^30^ suggesting that, as for influenza vaccines, YF‐17D‐induced accessory cell‐derived cytokines may also induce development of CIML NK cells. As in influenza vaccination, this pre‐activation state is short‐lived suggesting that there is no lasting imprint on the NK cell repertoire. These transient innate responses (including NK cells) may, however, synergise with antigen‐specific vaccine‐induced responses resulting in the formation of particularly durable and effective T cell‐ and B cell‐mediated immunity to YFV.^30^, ^65^ A more robust mechanistic understanding of the induction and function of CIML NK cells during infection or vaccination with YFV and other flaviviruses will help to define their role.

## Human immunodeficiency virus

HIV remains highly prevalent across the world with 2.1 million new infections estimated in 2015; lifelong treatment is required to prevent disease and death, which places a considerable burden on health systems worldwide.^66^ A prophylactic HIV vaccine is of utmost priority. HLA‐I and killer cell immunoglobulin‐like receptor (KIR) genotype and NK cell education influence killing of HIV‐1‐infected CD4^+^ T cells and are associated with the rate of progression of HIV infection.^67^, ^68^ In the partially successful RV144 vaccine trial, IgG against variable regions 1 and 2 of the HIV‐1 envelope glycoprotein was inversely correlated with the rate of infection.^69^ Indeed, RV144 induced isotypes IgG1 and IgG3 targeting the crown of the V2 loop demonstrating the potential for NK cell ADCC induction.^70^, ^71^ NK cells from KIR3DL1/HLA‐Bw4^+^ or KIR2DL1/HLA‐C2^+^ donors show higher cytotoxicity against HIV‐infected targets in the presence of anti‐HIV gp120 antibody, highlighting the influence of NK cell education to HIV vaccine‐induced effector NK cells and potentially contributing to individual variability in vaccine outcomes.^72^, ^73^ CD57^+^NKG2C^+^ memory‐like NK cells are expanded in HIV‐1/HCMV co‐infected individuals, and these cells make a potential contribution to control of viremia during primary HIV infection.^74^, ^75^ Together with evidence that individuals with a degree of inherent resistance to HIV – so‐called elite controllers or slow progressors – mount stronger antibody‐mediated NK cell activation and ADCC responses than more susceptible individuals, these studies suggest that NK cells may contribute to HIV protection and control.^76^

NK cells have been implicated as antigen‐specific effector cells after vaccination or infection of nonhuman primates with simian immunodeficiency virus (SIV); target cells pulsed with SIV vaccine antigen but not heterologous antigens can be lysed *in vitro* by splenic and hepatic NK cells from infected but not from uninfected animals.^17^ These antigen‐specific responses could be detected for at least 5 years after SIV DNA/adenovirus prime‐boost vaccination, suggesting that this memory‐like response is long‐lived.^17^ By contrast, no significant potentiation of circulating NK cell function was observed after SIV infection or vaccination; rather, SIV infection impaired the cytotoxic response of peripheral blood NK cells.^77^ However, a trend towards increasing *in vitro* NK cell CD107a expression in response to IL‐15 and IL‐12 postvaccination suggests that memory‐like NK cells with enhanced cytokine responsiveness may have been induced in this study.^77^

In HIV patients, therapeutic HIV vaccination or IL‐2 treatment sustains or enhances NK cell activity.^32^, ^78^ Immunisation of chronically infected patients with an adjuvanted HIV‐1 Gp120/NefTat subunit protein vaccine induces IL‐2 from T helper cells and an increase in NK cell IFN‐γ production *in vitro*; NK cell IFN‐γ production was reduced by depletion of CD4^+^ T cells and almost completely abrogated after blocking both IL‐2 and IL‐12, suggesting a role for accessory cells in full NK cell effector functions after vaccination.^32^ These, and other, studies highlight the potential of therapeutic vaccination to restore NK cell function during chronic HIV infection.^32^, ^79^

## Ebola

Several vaccines are in development for the prevention of Ebola virus disease (EVD). Two vectored vaccines that express the glycoprotein (GP) from the Zaire strain of Ebola (ZEBOV) and use the recombinant vesicular stomatitis virus and Chimp Adenovirus type 3 (rVSV‐ZEBOV and ChAd3‐ZEBOV, respectively) are the most advanced of these.^80^ Ebola virus has a wide range of host cell targets including macrophages and DCs, infection of which aids viral dissemination and crucially leads to immune dysregulation.^81^ Little is known about the role of NK cells in Ebola virus infection but *in vitro* studies show IFN‐inhibiting domains (IIDs) within Ebola viral proteins VP24 and VP35 interrupt DC maturation and type I IFN signalling leading to somewhat impaired NK cell activation and cytotoxicity.^82^ Disrupting either of these IIDs restores DC maturation and NK cell activation as measured by NKp46 and CD38 expression.^82^ Another study showed that Ebola virus‐like particles (VLPs) lacking IIDs activated NK cells and led to lysis of filovirus‐infected autologous human DCs in culture and pro‐inflammatory cytokine release.^83^

Activation of the early inflammatory response and release of cytokines such as IP‐10, IL‐1β, IL‐6 and TNFα, correlated with survival from EVD in humans^81^, ^84^ and mice can be protected against Ebola by adoptive transfer of NK cells from VP40 containing VLP‐treated mice.^85^ Increased survival of mice after postexposure vaccination with the candidate vaccine rVSV∆G‐EBOV is reversed by NK cell depletion;^86^ postexposure vaccination stimulated a burst of IFN‐γ release and type I IFN secretion from accessory cells, potentially kick‐starting the antiviral response and overcoming the blockade caused by IIDs.^86^ Postexposure antibody therapy has also been shown to give effective protection in animal models via ADCC activity.^87^, ^88^ These studies implicate NK cells as important effectors in protection against Ebola virus infection and in vaccine‐induced immunity and raise the potential of indirect cytokine activation of NK cells to restrict virus dissemination after therapeutic vaccination.

## Malaria

The role of NK cells in natural immunity or vaccine‐induced protection against malaria infection remains to be established.^89^ NK cell activation has been described to varying degrees in different experimental murine models^90^, ^91^ and NK cells have been shown to contribute directly to the elimination of *Plasmodium falciparum*‐infected red blood cells (RBC) in a humanised mouse model.^92^ *In vitro* studies of human PBMC show NK cells are readily activated by *P. falciparum*‐infected RBC; the resulting NK cell proliferation, IFN‐γ production, CD25 and CD69 expression were further demonstrated to be dependent on IL‐2 and accessory cell IL‐12 and IL‐18 production and on cell–cell contact.^93^, ^94^, ^95^, ^96^ In humans, long‐lasting NK cell activation has been reported in controlled human malaria infection (CHMI) studies; a decrease in peripheral blood NK cell frequency early after infection suggests migration of NK cells into the tissues, possibly the liver.^97^, ^98^, ^99^

RTS,S/AS01 is the most promising vaccine tested to date for human *P. falciparum* malaria. RTS,S consists of recombinant circumsporozoite surface protein (CSP) of *P. falciparum* fused to the hepatitis B virus surface antigen (HBs) and adjuvant delivery system (AS)01 formed into VLPs. PBMC collected from a RTS,S randomised controlled trial revealed postvaccination IL‐2 secretion with IFN‐γ and CD69 upregulation on NK cells in response to *in vitro* restimulation with HBs or CSP. All responses were significantly higher in RTS,S vaccines compared to control rabies vaccinated subjects.^31^ A weak association has been reported between IL‐2 secreting CD4^+^ T cells and time to parasitaemia, accompanied by an increase in the proportion of CD56^bright^ NK cells, higher IFN‐γ and perforin expression, and protection against malaria challenge in vaccine recipients has also been reported.^100^ Interestingly, peripheral blood NK cell gene expression signatures were negatively correlated with RTS,S‐induced malaria protection, consistent with migration of activated blood NK cells to the tissues,^101^ which implies that peripheral NK cell responses to malaria play a minimal role in vaccine responses.

## Tuberculosis

The live attenuated BCG vaccine is the only vaccine currently licenced for the prevention of tuberculosis disease (TB) caused by *Mycobacterium tuberculosis* (M.tb) and is administered to over 120 million infants each year.^102^ NK cells are an important component of the cellular immune response to BCG, producing more than half of the total IFN‐γ after vaccination in newborns and 2‐month‐old infants.^102^

BCG, and other live vaccines such as measles vaccine, have been shown to induce nonspecific effects that are beneficial to the recipient and reduce overall mortality in a community.^103^, ^104^ Potential underlying mechanisms include T cell‐mediated cross‐reactivity and/or ‘training’ or ‘priming’ of innate immune cells, including monocytes and NK cells. Increased expression of pattern recognition receptors (PRR) in monocytes, and higher levels of IFN‐γ, TNFα and IL‐1β secretion have been observed when PBMC from BCG‐vaccinated individuals are restimulated with mycobacterial or unrelated antigens, compared to prevaccination PBMCs.^22^ These effects persisted for up to 12 months after BCG vaccination and were partly attributed to epigenetic remodelling of key cytokine gene loci and have been termed ‘trained immunity’. Similarly, increased NK cell CD69 expression in response to Pam3Cys has been reported in post‐BCG vaccination samples from infants and correlated with higher concentrations of IL‐12 secretion.^105^ Interestingly, no changes in NK cell phenotype, maturation or IFN‐γ production were reported in BCG‐trained NK cells,^106^ suggesting that they are not equivalent to CIML NK cells.

Enhancement of NK cell IFN‐γ responses to BCG has been reported after BCG vaccination of patients with latent TB^29^ and in 5‐week‐old infants who were BCG‐vaccinated at birth compared to unvaccinated controls;^29^ NK cell responses were completely abrogated by neutralisation of IL‐12 and IL‐18.^29^ Consistent with studies of other whole organism vaccines, as described above, these studies indicate that enhanced responsiveness to cytokines is a key feature of vaccine‐mediated effects on NK cells.

## The role of vaccine adjuvants in promoting NK cell responses

Killed whole organism or live attenuated vaccines are both highly immunogenic and particularly effective at potentiating NK cell responses; both of these traits likely reflect the presence of potent pathogen‐associated molecular patterns (PAMPs) for PRR‐mediated accessory cell activation. PAMP‐containing adjuvants are typically required to improve the immunogenicity of subunit or vectored vaccines, which lack these ligands. Several studies have documented enhancement of NK cell activation by adjuvants.^32^, ^107^, ^108^ IL‐15‐matured DCs exposed *in vitro* to the TLR‐4 agonist AS04‐adjuvanted human papilloma virus (HPV) VLP vaccine can potentiate NK cell activation and killing of HPV‐infected cells compared to either IL‐4‐matured DCs or VLP alone; this effect was attributed to the superior cytokine‐producing ability of the DCs.^109^ Similarly, vaccination in the presence of exogenous IL‐15 enhances DC maturation and protection against lethal staphylococcal enterotoxin B challenge in mice compared to vaccine alone.^110^

AS03, a squalene‐based adjuvant, promotes recruitment of antigen‐presenting cells (APCs) and antigen processing. A system‐wide analysis of the response to AS03‐adjuvanted inactivated H5N1 influenza vaccine revealed a direct correlation between IP‐10, type I and II interferon production, and enhanced NK cell activation and proliferation.^111^, ^112^ Similarly, a bursin‐like peptide shown to stimulate immune cells induced higher levels of IL‐2 and IL‐4 and increased NK cell frequencies and IFN‐γ secretion in mice vaccinated with inactivated influenza H9N2 compared to vaccine alone.^113^ Taken together, these studies indicate that PRR‐mediated activation and maturation of accessory cells such as DCs by vaccine adjuvants increase the production of costimulatory cytokines leading to heightened NK cell activation. Whether these NK cells share features of CIML NK cells has not yet been formally tested.

## Concluding remarks

**Figure 1:**
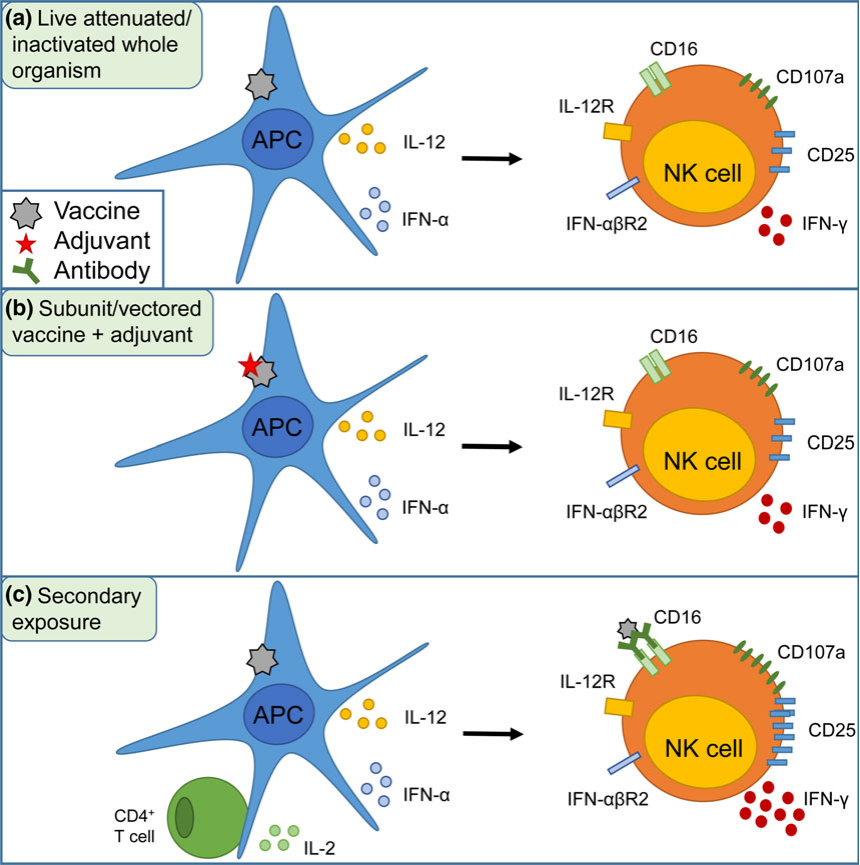
Accessory cell‐dependent NK cell activation after vaccination. **(a)** Activation of APCs by live attenuated or inactivated whole organism vaccines induces the release of costimulatory cytokines which in turn leads to NK cell activation including IFN‐γ release, degranulation and CD25 upregulation. **(b)** Adjuvants promote accessory cell function for subunit or vectored vaccines in the absence of vaccine‐derived PAMPs. **(c)** Upon secondary exposure, IL‐2 from memory CD4+ T cells, antibody and the presence of CIML NK cells enable an enhanced response.

Although there is now considerable evidence of enhanced NK cell responses after vaccination, the functional importance of NK cells in vaccination‐induced immunity is rather difficult to evaluate. The NK cell response to vaccination varies depending on the type of vaccine, the cytokine signature induced by the vaccine/adjuvant combination and subsequent accessory cell activation (Figure 1). The ability of NK cells to respond to signals from both innate and adaptive immune cells suggests that when one arm of the immune response is impaired, such as T cell responses in HIV infection or innate cell dysregulation in EVD, NK cells may play an important immune effector role, maximising the impact of the remaining arm of the immune system. Successful activation of APCs and induction of an early inflammatory response by a vaccine correlate with enhanced and sustained NK cell activation and function. Importantly, NK cell education by HLA‐KIR or other receptor‐ligand combinations may well calibrate functional capacity on induction by both adaptive and innate pathways thereby driving individual variability in vaccine‐induced responses. The addition of adjuvant systems to vaccines to increase accessory cell activation and therefore augmenting NK cell function including ADCC activity could play a role in the future design of new vaccines, postexposure therapy, therapeutic cancer vaccines, regimen optimisation and evaluation of vaccine efficacy.
